# Pure large cell neuroendocrine carcinoma of the bladder without urological symptoms

**DOI:** 10.11604/pamj.2018.30.134.13437

**Published:** 2018-06-14

**Authors:** Ekrem Akdeniz, Mustafa Bakirtas, Mustafa Suat Bolat, Sevda Akdeniz, Ismail Özer

**Affiliations:** 1 Health Sciences University, Samsun Training and Research Hospital, Department of Urology, Samsun, Turkey; 2 Health Sciences University, Samsun Training and Research Hospital, Department of Pathology, Samsun, Turkey; 3 Health Sciences University, Samsun Training and Research Hospital, Department of Anesthesiology and Reanimation, Samsun, Turkey; 4 Health Sciences University, Samsun Training and Research Hospital, Department of Nephrology, Samsun, Turkey

**Keywords:** Bladder, neuroendocrine carcinoma, tumor

## Abstract

Neuroendocrine carcinoma is one of the uncommonly seen pathologies of the urinary bladder. Macroscopic hematuria is frequently encountered symptom in patients with neuroendocrine carcinoma. We report a 45-year-old man with left solitary kidney and oliguria for five days the development of acute renal failure (ARF) with the impaired general condition. The underlying cause being identified as pure type large-cell neuroendocrine carcinoma of the bladder. Large-cell neuroendocrine carcinoma of the bladder is an uncommon fatal tumor. No macroscopic hematuria or urological symptom was observed in our case. Advanced ectasia was not observed in the kidney, and the patient's clinical status was complicated with ARF. It must not be forgotten that in some bladder tumors, the patient's general condition may be impaired without urological symptoms.

## Introduction

Neuroendocrine tumors originating from the enterochromaffin cell-rich epithelium are generally seen in the gastrointestinal system. Enterochromaffin cells are also present, albeit in low numbers, in the prostate and the urinary bladder and neuroendocrine tumors can also be seen in the urinary system [1]. Neuroendocrine tumors constitute approximately 1% of bladder tumors. There are two subtypes, small- and large-cell. The small-cell type is by far the most common form, and fewer than 30 large-cell neuroendocrine carcinomas (LCNC) have to date been reported in the English language literature [2,3]. No explicit treatment protocol has yet been described for LCNC. The prognosis is quite poor, and the outcomes are fatal [3]. We report the clinical findings of a 45-year-old man diagnosed with LCNC.

## Patient and observation

**Figure 1:**
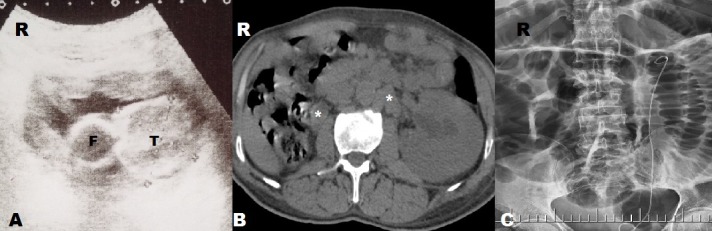
Radiological findings: A) bladder ultrasonography T: tumor; F: Foley catheter; B) non-contrast-computed tomography image, lymph node swelling (asterisks); C) JJ stent after transurethral resection

**Figure 2:**
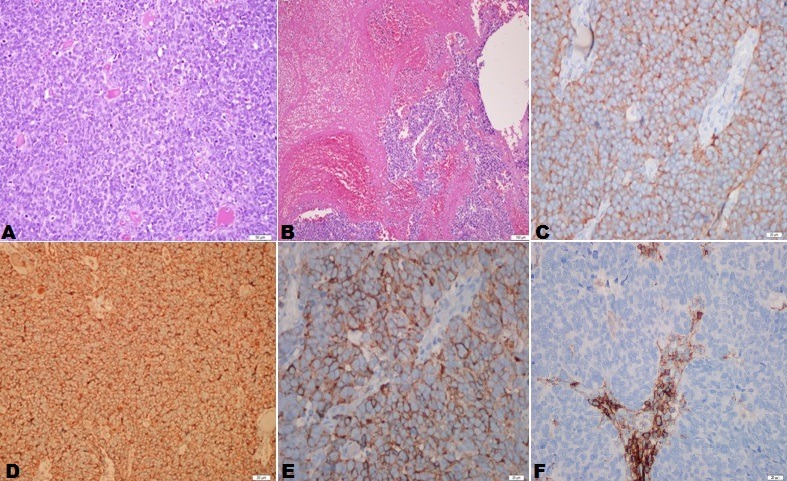
Histological examination: tumoral tissue with intense cellularity in a loose vascularized stroma, hematoxylin-eosin staining, x200; B) diffuse areas of necrosis and hemorrhage in the tumor, x100; C) synaptofizin positive, x400; D) chromogranin A positive, x200; E) CD56 positive, x400; F) CD44 negative, x400)

**Figure 3:**
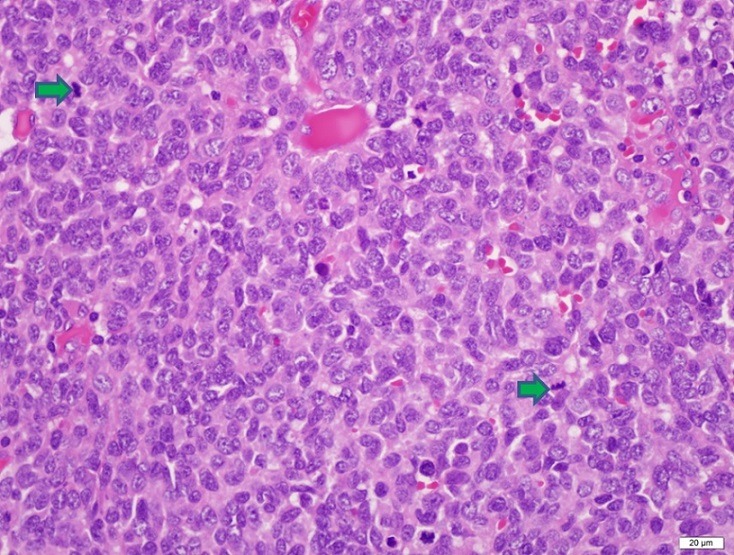
Large numbers of atypical mitotic figures in all fields (indicated by an arrow), hematoxylin-eosin staining, x400

A 45-year-old patient with congenital left solitary kidney presented to the emergency department due to the poor general condition. His urine output had decreased gradually over the previous 5 days. No gross hematuria or any urological symptoms (pollakiuria, dysuria, urgency etc.) were present. His serum blood urea nitrogen level was 28 mg/dL, creatinine 21.8 mg/dL, and potassium 6.8 mg/dL. Non-contrast computerized tomography (CT) and ultrasonography were performed under emergency conditions due to acute renal failure. Mild dilatation was observed in the left kidney and an approximately four cm mass in the left lateral wall of the bladder were observed at CT (Figure 1). The patient was taken for emergency hemodialysis. Cystoscopy was performed when his general condition improved. Cystoscopy revealed a necrotic solid mass, approximately 4 cm in diameter, in the left lateral bladder wall. The mass was approximately 2 cm distant from the left orifice, which was normal. The lesion in the bladder was transurethrally resected, and a JJ stent was inserted in the left kidney. Following JJ stent insertion, serum biochemical values returned to normal limits and no further hemodialysis was required. The tumor pathology was reported as pure type LCNC. Histological examination revealed large tumor cells with polymorphic nuclei and organoid, trabecular growth, and a low nucleus/cytoplasm ratio, a coarse chromatin pattern, prominent nucleoli, a high mitotic rate and necrosis in the cells. Immunohistochemically, the tumor neuroendocrine tumor differentiation markers CD56, chromogranin A and synaptophysin were positive while CD44 was negative, exhibiting neuroendocrine differentiation (Figure 2, Figure 3). No evidence of tumor was observed at CT of the chest, abdomen and pelvis. Treatment plans and alternatives were discussed with the patient. A combination of four courses of carboplatin/etoposide chemotherapy and radiotherapy to the pelvis and bladder region was scheduled. The patient was monitored with cystoscopic controls. An advanced surgical approach such as cystectomy was not considered.

## Discussion

Large-cell neuroendocrine tumors of the urinary bladder are uncommon. There have been very few publications concerning LCNC, and since these have generally been in the form of case reports, this prevents sufficient information being obtained to establish a standard algorithm for use in the diagnosis, treatment and monitoring of LCNC, resulting in difficulties for both patients and physicians. In clinical terms, lower urinary system symptoms such as hematuria (68.2%), dysuria and obstructive voiding symptoms are observed in small-cell neuroendocrine tumors [4,5]. In a review assessing patients with pure type LCNC, Pusiol et al reported that hematuria was present in 8 out of 11 patients with reported clinical symptoms [6]. Hematuria and lower urinary system symptoms did not develop in our patient. However, ureteral obstruction, another urinary pathology, did develop, and postrenal acute kidney failure developed in association with this. Neuroendocrine tumors are generally seen in the elderly and at more advanced clinical stages. Smoking is the most important risk factor [7]. Although various theories have been proposed concerning the tumor formation, the most widely accepted is the multipotent stem cell theory. According to this theory, multicentric cancer cells develop with changes in carcinogenic process in the bladder and studies have revealed that this is closely associated with smoking [1]. No history of smoking was present in our patient. There is no standardized treatment protocol for local or metastatic disease in neuroendocrine tumors of the bladder [3]. Treatment is modeled on pulmonary small-cell neuroendocrine carcinoma. Combinations of chemotherapy, radiotherapy and surgery are used, and cisplatin-based chemotherapy represents the basis of treatment [1]. There is uncertainty concerning what form of surgery should be performed in these cases. Cheng et al. reported no difference between a group undergoing cystectomy in small-cell carcinomas of the bladder and a non-cystectomy group [8]. However, that research involved small-cell carcinomas. Due to the low numbers of LCNC patients, description of treatment in the literature remains at the case report level. Hata et al treated patients with LCNC using transurethral resection alone and observed no recurrence at the postoperative 8^th^ month [9]. Dowd et al applied transurethral resection + radiotherapy + chemotherapy to patients with poorly differentiated neuroendocrine tumor and observed no recurrence in the 1^st^ year postoperatively [3]. Colarossi et al treated LCNC patients using a combination of cystectomy + hysterectomy + lymphadenectomy + chemotherapy and reported that patients died in the 7^th^ month postoperatively [10]. Although chemotherapy + radiotherapy was planned in our case, we decided to monitor our patient with cystoscopy. General survival for LCNC, with its aggressive course, has been reported at less than 2 years for metastatic disease and less than 1 year for metastatic disease [7]. As with treatment, there is also still no protocol for monitoring in LCNC, and the disease is monitored in the same way as other urothelial cell carcinomas [3]. Cystoscopy is particularly important and must be performed every 3 months. Because this tumor is prone to microscopic metastases, radiological imaging of the upper and lower urinary tract is essential. Metastasis to the brain is more common in neuroendocrine tumors of the bladder compared to other forms of urothelial cancers, and brain metastasis in small-cell neuroendocrine cancers is reported close to 11% [7]. Cerebral MRI is required in the monitoring of these patients.

## Conclusion

The data for LCNC consist solely of case reports. It is impossible to determine the prognosis of the disease or how it should be treated and follow-up with such limited information. Although prospective studies with large patient numbers are needed for standard therapeutic and follow-up protocols, the limited information available for LCNC makes even case reports valuable. Increasing the numbers of these reports will at least help clinicians produce a road-map.

## Competing interests

The authors declare no competing interest.
